# A new species of *Kurixalus* from western Yunnan, China (Anura, Rhacophoridae)

**DOI:** 10.3897/zookeys.770.23526

**Published:** 2018-07-04

**Authors:** Guohua Yu, Hong Hui, Dingqi Rao, Junxing Yang

**Affiliations:** 1 State Key Laboratory of Genetic Resources and Evolution, Kunming Institute of Zoology, Chinese Academy of Sciences, 32 Jiaochang Donglu, Kunming, Yunnan 650223, China

**Keywords:** China, *Kurixalus yangi* sp. n., new species, Western Yunnan

## Abstract

A new species of the genus *Kurixalus* (Anura: Rhacophoridae) is described from western Yunnan, China. Genetically the new species, *Kurixalus yangi* **sp. n.**, is closer to *Kurixalus naso* than to other known congeners. Morphologically the new species is distinguished from all other known congeners by a combination of the following characters: smaller ratios of head, snout, limbs, IND, and UEW to body size; male body size larger than 30 mm; curved canthus rostralis; weak nuptial pad; brown dorsal color; absence of large dark spots on surface of upper-middle abdomen; presence of vomerine teeth; gold brown iris; single internal vocal sac; serrated dermal fringes along outer edge of limbs; granular throat and chest; rudimentary web between fingers; and presence of supernumerary tubercles and outer metacarpal tubercle.

## Introduction

**Figure 1.**
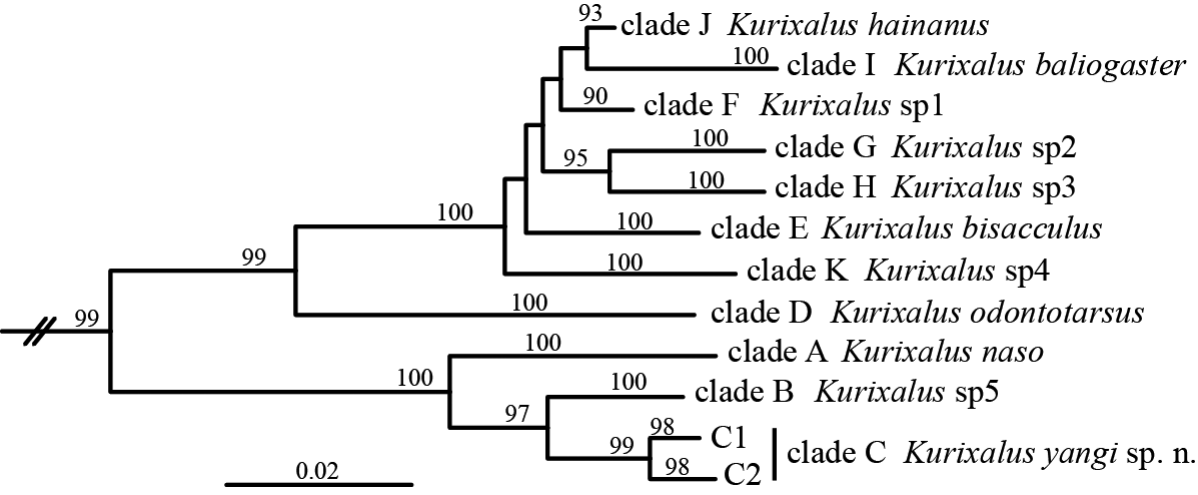
Simplified Neighbor-joining tree of the *Kurixalus odontotarsus* species group reproduced from Yu et al. (2017a).

The genus *Kurixalus* Ye, Fei, & Dubois in Fei (1999) distributes widely in eastern India, Indochina, Sunda Islands, Philippine archipelago, montane forests of southern China, and adjacent continental islands, and currently contains 15 species (Frost 2018). Owing to its morphological conservativeness, the taxonomy and systematics of *Kurixalus* were once very confusing (Yu et al. 2017a). For instance, *Kurixalus hainanus* (Zhao, Wang, & Shi in Zhao et al. 2005) was once thought to be a synonym of *Kurixalus odontotarsus* (Ye & Fei in Ye et al. 1993) by some authors (e.g., Fei et al. 2010) or a synonym of *Kurixalus bisacculus* (Taylor, 1962) by Yu et al. (2010). On the basis of broad sampling, recently Yu et al. (2017a) suggested that *K. hainanus* is valid and revealed six lineages that might represent undescribed species in the genus *Kurixalus*, one of which occurs in western Yunnan, China and northern Myanmar and is genetically closer to *Kurixalus naso* (Annandale, 1912) than to other known congeners with a divergence of 6.18% estimated from COI sequences (clade C, Fig. 1).

Here we further describe the lineage consisting of specimens from western Yunnan, China as a new species. Morphological comparisons demonstrate that the new species is distinctive from *K. naso* and other known congeners and therefore warrants taxonomic recognition.

## Materials and methods

**Figure 2.**
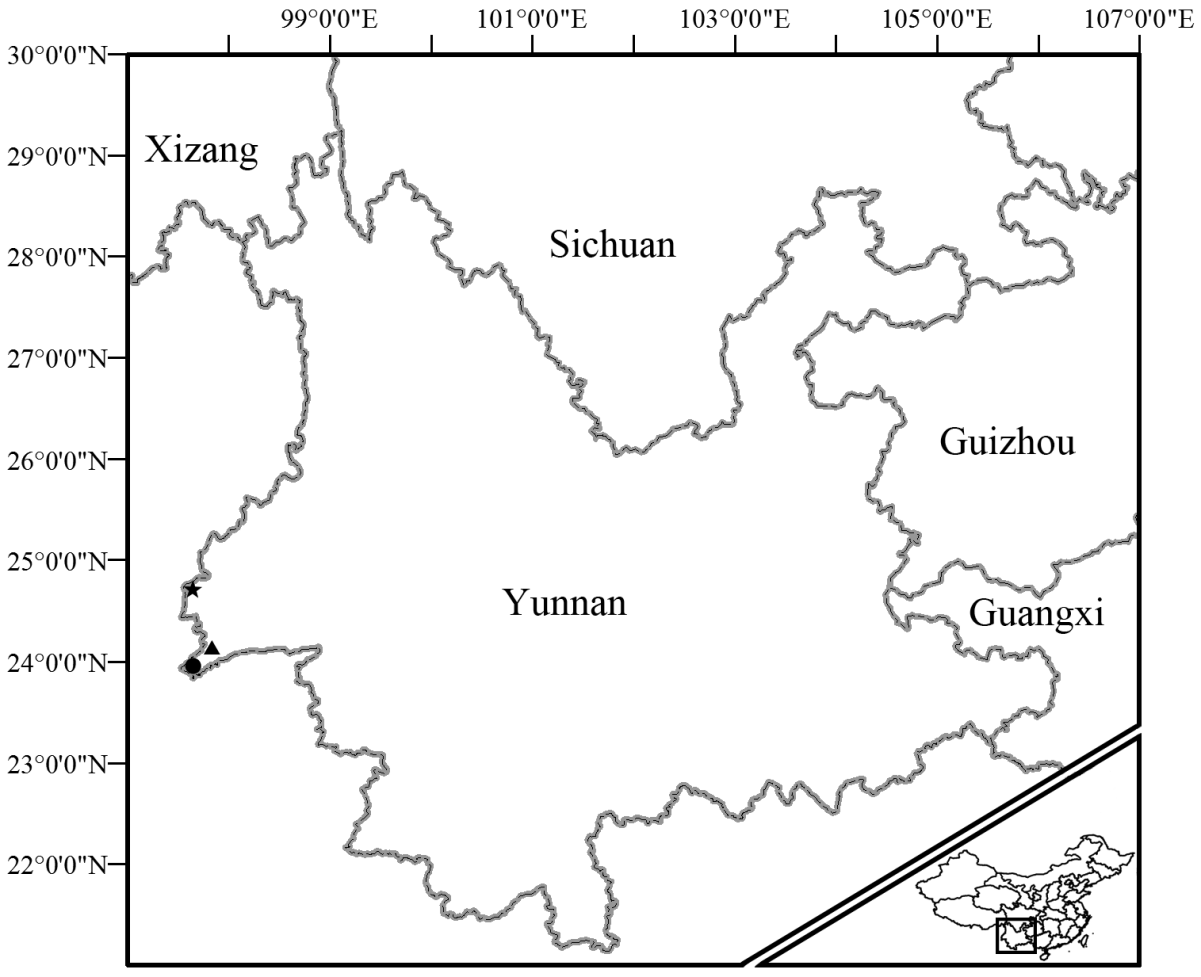
Collection sites of *Kurixalus yangi* sp. n. from western Yunnan, China. Star indicates the type locality, triangle indicates Nanjingli Village, and circle indicates Dengga Village.

*Sampling*. Specimens were collected during fieldwork in Dehong Autonomous Prefecture, western Yunnan, China in June and July, 2014 (Fig. 2). They were euthanized with diethyl ether anesthesia and fixed by 90% ethanol before being stored in 70% ethanol. Liver tissues were preserved in 99% ethanol. Specimens were deposited at Kunming Institute of Zoology, Chinese Academy of Sciences.

*Morphology*. Morphometric data were taken using digital calipers to the nearest 0.1 mm. Morphological terminology follows Fei (1999). Measurements include:

**SVL** snout-vent length (from tip of snout to vent);

**HL** head length (from tip of snout to rear of jaws);

**HW** head width (width of head at its widest point);

**SL** snout length (from tip of snout to anterior border of eye);

**IND** internarial distance (distance between nares);

**IOD** interorbital distance (minimum distance between upper eyelids);

**UEW** upper eyelid width (maximum width of upper eyelid);

**ED** eye diameter (diameter of exposed portion of eyeball);

**TD** tympanum diameter (the greater of vertical or horizontal diameter of tympanum);

**DNE** distance from nostril to eye (from posterior border of nostril to anterior border of eye);

**FLL** forelimb length (distance from elbow to tip of third finger);

**THL** thigh length (distance from vent to knee);

**TL** tibia length (distance from knee to heel);

**FL** foot length (distance from proximal end of inner metatarsal tubercle to tip of fourth toe);

**TFL** length of foot and tarsus (distance from tibiotarsal joint to tip of fourth toe).

A multivariate principal component analysis (PCA) was conducted using SPSS 17.0 (SPSS Inc.) based on a correlation matrix of size-standardized measurements (all measurements divided by SVL). Scatter plots of the scores of the first two factors of the PCA were used to examine the differences between the new species and *K. naso*. Additionally, the differences between the new species and its two congeners known from Yunnan, China (*K. odontotarsus* and *K. hainanus*) were also similarly examined based on morphometric data.

## Results

**Figure 3.**
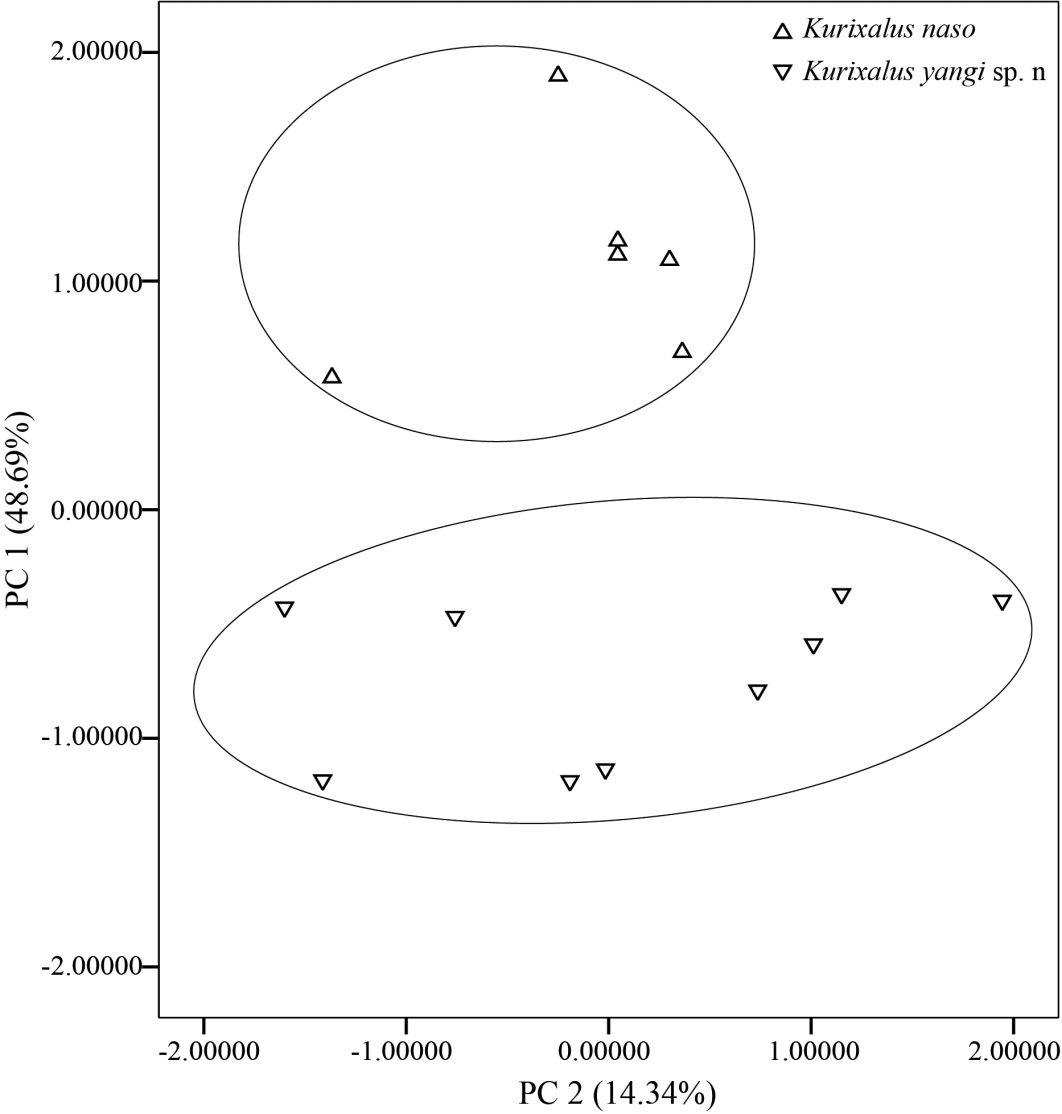
Scatterplot of principal components 1 and 2 of size-adjusted morphometric data for males of *Kurixalus yangi* sp. n and *Kurixalus naso*.

**Figure 4.**
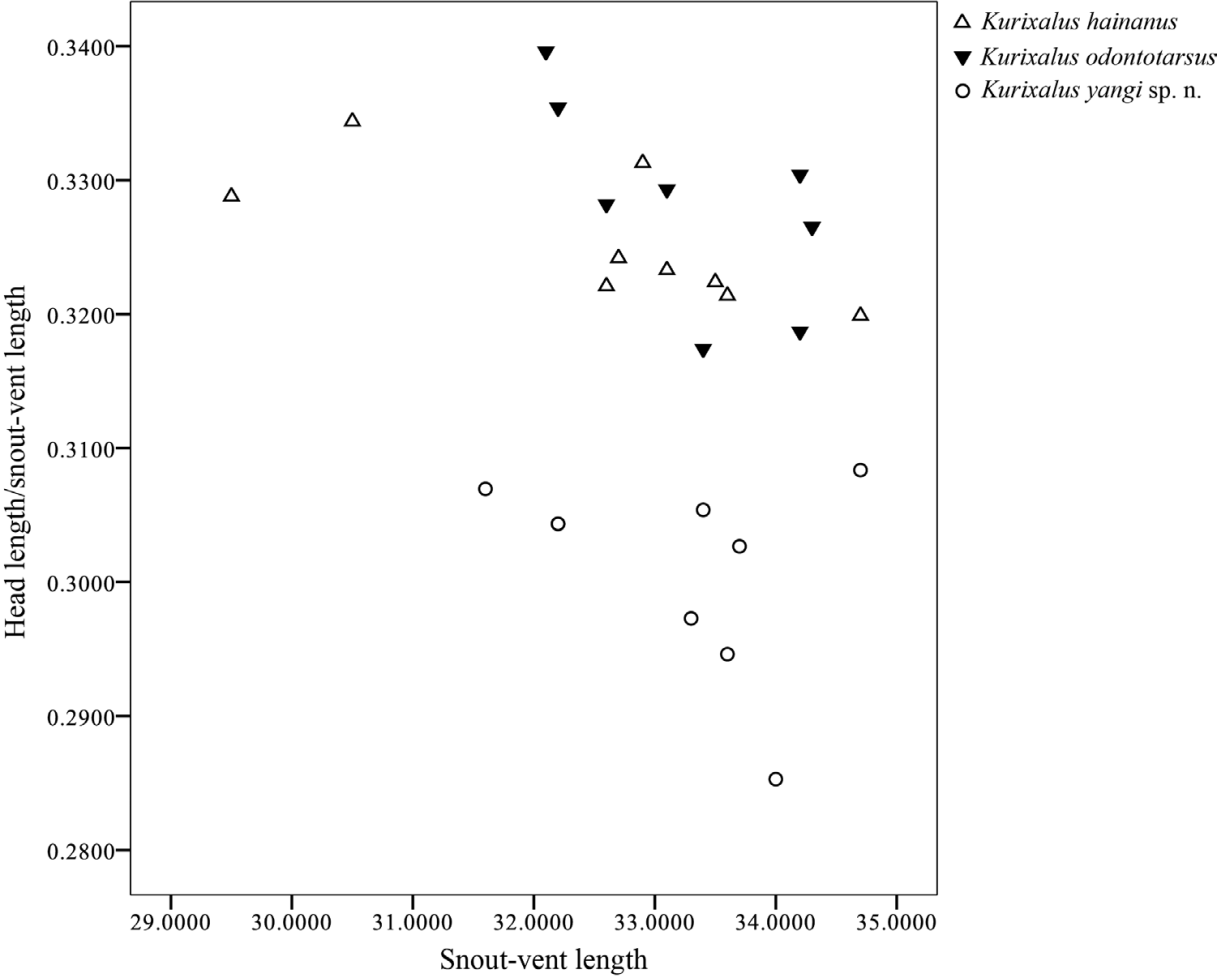
Scatterplot of ratio of head length against snout-vent length for males of *Kurixalus yangi* sp. n., *Kurixalus hainanus*, and *Kurixalus odontotarsus*.

Morphometric data of the new species and *K. naso* are summarized in Table 1. We retained the first two principal components which accounted for 63.03% of the total variance and had eigenvalues above 2.0 (Table 2). Loadings for PC 1, which accounted for 48.69% of the total variance, were all positive except for TD and were most heavily loaded on HL, SL, IND, UEW, FLL, THL, TL, and TFL (Table 2). Differentiation was found along the PC 1 axis between *K. naso* and the new species (Fig. 3). This result indicates that the new species differs from *K. naso* by a series of characters associated with the head and limbs such as shorter HL, shorter SL, narrower IND, narrower UEW, shorter FLL, shorter THL, shorter TL, and shorter TFL. The second principal component (PC 2) accounted for 14.34% of the total variance and loaded heavily and positively on IOD and negatively on HW (Table 2), but no clear separation was observed along this axis between the new species and *K. naso* (Fig. 3). In addition, the new species can be separated from *K. odontotarsus* and *K. hainanus* by having smaller ratio of head length to body size (Fig. 4).

**Table 1.** Measurements of *Kurixalus yangi* sp. n. and *Kurixalus naso*. Abbreviations defined in text.

	Voucher no	SVL	HL	HW	SL	IND	IOD	UEW	ED	TD	DNE	FLL	THL	TL	TFL	FL
*Kurixalus yangi* sp. n.	KIZ 14102901	32.2	9.8	11.7	4.6	3.2	3.4	2.9	4.0	2.1	2.5	15.5	15.3	15.6	22.0	14.3
	KIZ 14102902	33.4	10.2	11.9	4.9	3.2	3.4	2.9	3.9	2.3	2.6	16.5	15.9	16.8	23.3	15.1
	KIZ 14102904	33.7	10.2	12.0	4.6	3.0	3.3	2.8	4.6	2.0	2.6	16.7	15.8	15.9	22.1	14.2
	KIZ 14102905	34.7	10.7	12.7	5.0	3.2	3.5	3.3	4.6	2.6	2.4	16.5	15.9	16.7	23.1	14.5
	KIZ 14102906	31.6	9.7	11.7	4.4	3.0	3.1	3.0	4.3	2.2	2.4	15.5	14.9	14.2	20.4	13.0
	KIZ 14102908	34.0	9.7	12.2	4.7	3.3	3.4	2.6	4.5	2.0	2.4	16.7	16.4	16.6	22.9	15.3
	KIZ 14102911	32.2	9.8	11.6	4.8	3.2	3.3	2.8	3.9	1.9	2.4	15.8	15.5	16.0	21.9	14.4
	KIZ 14102912	33.3	9.9	12.1	4.8	3.1	3.1	3.3	4.4	2.0	2.5	16.7	16.2	16.4	21.8	14.7
	KIZ 14102913	33.6	9.9	12.3	4.4	3.3	2.8	3.5	4.2	1.6	2.6	16.4	16.3	16.7	22.5	14.8
*Kurixalus naso*	KIZ 180001R	31.6	10.3	11.3	5.0	3.3	3.2	3.4	4.3	1.6	2.1	15.9	16.1	16.0	21.7	13.9
	KIZ 180002R	31.9	10.1	11.9	5.1	3.2	2.9	3.1	4.3	1.4	2.4	16.1	15.8	16.2	22.0	14.1
	KIZ 180003R	32.5	11.1	12.0	5.3	3.7	3.2	3.7	4.7	2.1	2.5	17.7	16.5	16.5	23.2	15.4
	KIZ 180004R	30.9	10.5	11.3	4.8	3.3	3.0	3.3	4.0	1.7	2.4	16.0	15.6	15.7	21.5	14.4
	KIZ 180005R	31.4	10.5	11.3	5.1	3.4	3.1	3.3	4.3	1.8	2.3	16.1	16.1	16.2	21.8	13.9
	KIZ 180006R	29.3	10.3	10.6	4.8	3.2	3.0	3.2	4.0	1.7	2.4	15.0	15.1	14.7	20.2	12.7

**Table 2.** Factor loadings of the first two principal components of 14 size-adjusted morphometric characteristics of males of *Kurixalus yangi* sp. n. and *Kurixalus naso*. Absolute values of loading greater than 0.70 in boldface. Abbreviations defined in text.

Character	PC 1	PC 2
Eigenvalue	6.817	2.008
% variation	48.691	14.339
HL	**0.866**	0.000
HW	0.187	-**0.706**
SL	**0.885**	0.135
IND	**0.947**	0.069
IOD	0.001	**0.776**
UEW	**0.783**	-0.384
ED	0.423	-0.486
TD	-0.388	0.367
DNE	0.182	-0.094
FLL	**0.882**	-0.111
THL	**0.927**	-0.050
TL	**0.788**	0.251
FTL	**0.827**	0.413
FL	0.658	0.330

### Kurixalus yangi sp. n.

Taxon classificationAnimaliaORDOFAMILIA

http://zoobank.org/DBB82038-0DCD-48E0-AADB-FE66C31B1A7A

Figs 5 , 6 , 7

#### Holotype.

KIZ 14102911, an adult male, collected at 21:10 on 30 June 2014 by Hong Hui from Nabang (24°46'12.03"N, 97°34’28.03"E, 354 m elevation; Fig. 2), Yingjiang County, Dehong Autonomous Prefecture, Yunnan, China.

#### Paratype.

Eight adult males: KIZ 14102901 and KIZ 14102902 collected at 20:40 on 10 July 2014 by Hong Hui from Dengga Village (23°59'21.05"N, 97°35'13.03"E, 868 m elevation; Fig. 2), Longdao Township, Ruili City, Dehong Autonomous Prefecture, Yunnan, China; KIZ 14102904–14102906 collected at 21:10 on 9 July 2014 by Hong Hui from Nanjingli Village (24°05'52.07"N, 97°50'30.07"E; 1366 m elevation; Fig. 2), Ruili City, Dehong Autonomous Prefecture, Yunnan, China, and KIZ 14102908, KIZ 14102912, and KIZ 14102913 collected at 21:10 on 30 June 2014 by Hong Hui from the type locality.

#### Etymology.

The species name is dedicated to Professor Datong Yang from Kunming Institute of Zoology, Chinese Academy of Sciences for his outstanding contribution to herptofauna research of Yunnan, China.

#### Diagnosis.

The new tree frog species is assigned to the genus *Kurixalus* based on a combination of the following characters: tips of digits enlarged to discs, bearing circum-marginal grooves; small body size (SVL range of 31.6–34.7 mm in adult males; Table 1); finger webbing poorly developed and toe webbing moderately developed; serrated dermal fringes along outer edge of forearm and tarsus; an inverted triangular-shaped dark brown mark between eyes; dorsal brown “) (” saddle-shaped marking; and coarse dorsal and lateral surfaces with small, irregular tubercles (Nguyen et al. 2014a, Nguyen et al. 2014b, Yu et al. 2017b). Our previous molecular study placed the new species in *Kurixalus* with other known congeners (Yu et al. 2017a).

*Kurixalus yangi* sp. n. can be distinguished from its congeners by a combination of the following characters: male body size larger than 30 mm; smaller ratio of head length to body size; curved canthus rostralis; weak nuptial pads; brown dorsal color; absence of large dark spots on upper-middle abdomen; presence of vomerine teeth; gold brown iris; single internal vocal sac; serrated dermal fringes along outer edge of limbs; granular throat and chest; interorbital space longer than upper eyelid; rudimentary web between fingers; and presence of supernumerary tubercles and thenar tubercle.

**Figure 5.**
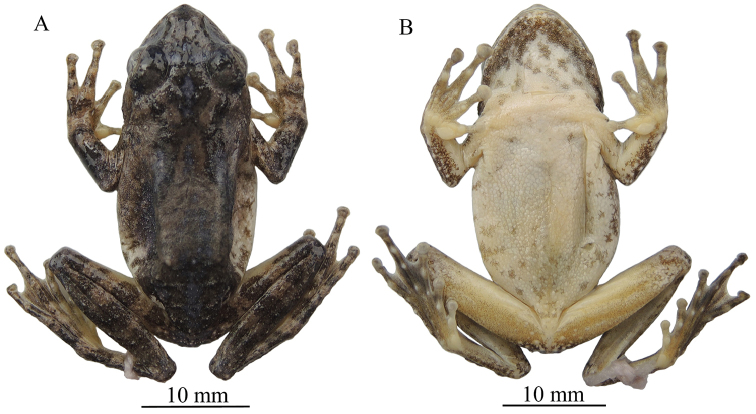
Dorsal (**A**) and ventral (**B**) views of the holotype of *Kurixalus yangi* sp. n. in preservative.

**Figure 6.**
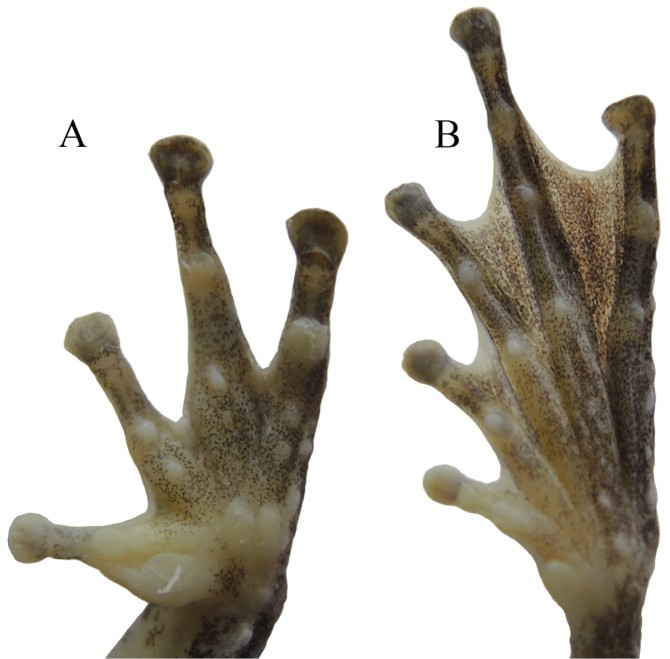
Ventral view of hand (**A**) and foot (**B**) of the holotype of *Kurixalus yangi* sp. n. in preservative.

**Figure 7.**
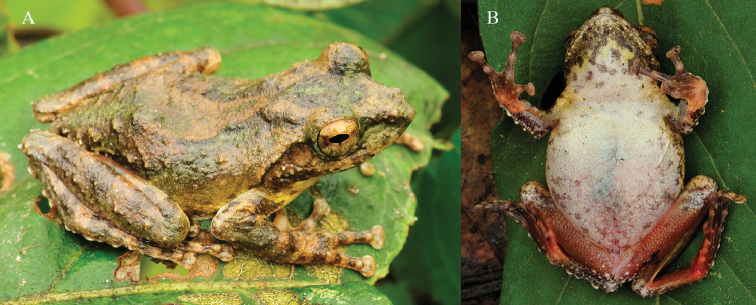
lateral-dorsal (**A**) and ventral (**B**) views of the holotype of *Kurixalus yangi* sp. n. in life.

#### Description of holotype.

A small rhacophorid; HL shorter than HW; snout pointed, no dermal prominence on tip, projecting beyond margin of lower jaw in ventral view; canthus rostralis blunt and curved; lore region oblique, slightly concave; nostril oval, slightly protuberant, closer to tip of snout than eye; IND slightly narrower than IOD; pineal spot absent; pupil oval, horizontal; tympanum distinct, rounded, slightly less than half ED; supratympanic fold distinct, curving from posterior edge of eye to insertion of arm; vomerine teeth in two oblique patches, touching inner front edges of oval choanae; tongue notched posteriorly; single internal vocal sac.

Relative length of fingers is I < II < IV < III. Tips of all four fingers expanded into discs with circum-marginal and transverse ventral grooves; relative width of discs is I < II < IV < III; nuptial pad present on first finger; fingers weakly webbed at base; lateral fringes on free edges of all fingers; subarticular tubercles prominent and rounded, formula 1, 2, 2, 1; supranumerary tubercles present; two metacarpal tubercles present; series of white tubercles forming serrated fringe along outer edge of forearm.

Heels overlapping when legs at right angle to body; relative length of toes is I < II < III < V < IV; tips of toes expanded into discs with circum-marginal and transverse ventral grooves; toe discs smaller than finger discs; relative size of discs is I < II < III < V < IV; webbing moderate on all toes, webbing formula is I1.5–2II1–2III1–2IV2–1V following Myers and Duellman (1982); subarticular tubercles prominent and rounded, formula 1, 1, 2, 3, 2; supernumerary tubercles present; inner metatarsal tubercle distinct, oval; outer metatarsal tubercle absent; series of tubercles forming serrated dermal fringe along outer edge of tarsus and fifth toe.

Numerous small to large tubercles scattered on top of head, upper eyelids, dorsum, and flanks; patch of white tubercles below vent; white tubercles on tibiotarsal articulation; throat and chest finely granulated and abdomen coarsely granulated; dorsal surface of limbs smooth with tuberculs and ventral surface of thighs granulated.

#### Color of holotype in life.

Iris golden brown; dorsal surface brown, mottled with green patches and a dark brown saddle-shaped mark on dorsum behind eye; a dark brown inverted triangular-shaped mark between eyes, posterior of which extends to and touches the saddle-shaped mark; lateral head and tympanic region brown, mottled with green patches below canthus and dark brown spots on edge of upper jaw; flank light yellow, mottled with green and brown patches; limbs dorsally brown with three clear dark brown bands, mottled with green; palm of hand light red; rear, anterior, and venter of thigh red; inner side of tarsus and foot red; chest and abdomen white, fringed with yellow and mottled with small brown spots; chin clouded with dark brown and mottled with yellow patches.

#### Color of holotype in preservative.

In preservative, green, yellow, and red faded. Dorsal ground color brown, pattern same as in life. Flank white with brown patches; margin of lower jaw clouded with dark brown; chin, chest, and abdomen white with scattered brown spots; palm of hand dirty white; anterior, posterior, and venter of thigh dirty white, with many fine brown speckling scattered on venter of thigh; inner side of tarsus and foot dirty white.

#### Variations.

Because the holotype and paratypes of the new species are all male, sexual dimorphism could not be determined. IND is smaller than IOD in holotype and most paratypes, but IND is larger than IOD in paratype KIZ 14102913 (Table 1). In addition, IOD is larger than UEW in holotype and most paratypes, but IOD is smaller than UEW in paratypes KIZ 14102912 and KIZ 14102913 (Table 1). Additionally, color pattern of paratype KIZ 14102912 also differs from other specimens in that its chin has much less spotting.

#### Distribution and natural history.

**Figure 8.**
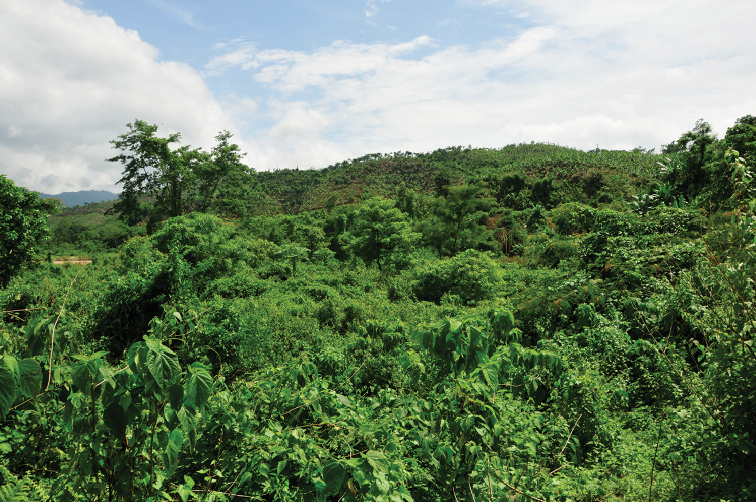
Habitat of *Kurixalus yangi* sp. n. at the type locality.

The new species is known from border region with northern Myanmar in western Yunnan, China (Fig. 2) and northern Myanmar according to Yu et al. (2017a). At the type locality, the new species was found calling on leaves of bushes adjacent to a road at night (Fig. 8). Specimens from the other two sites were found calling on broad leaves at the edge of an evergreen forest. Tadpoles, eggs and females were not found.

#### Comparisons.

The new species, *Kurixalus yangi* sp. n., is genetically closer to *K. naso* than to other known members of *Kurixalus* according to our previous work (Yu et al. 2017a), but morphologically it can be separated from *K. naso* by having smaller ratios of head, snout, IND, UEW, and limbs divided by SVL (Table 2 and Fig. 3). The smaller IND and UEW ratios in the new species can be observed when comparing these distances with the IOD, which is generally larger in the new species but smaller in *K. naso* (Table 1).

**Figure 9.**
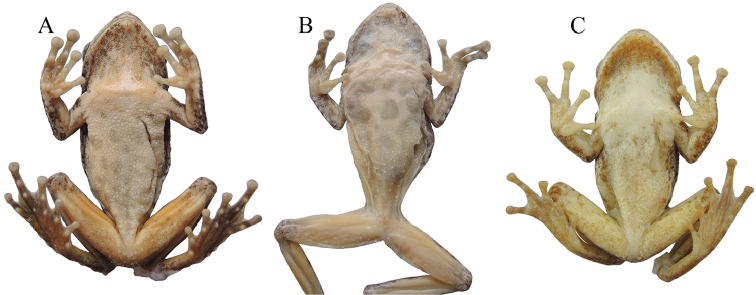
Ventral views of **A** *Kurixalus odontotarsus* (KIZ 180019Y) **B** *Kurixalus hainanus* (KIZ 180007R), and **C** *Kurixalus lenquanensis* (KIZ 170184Y; from Yu et al. 2017b).

Currently, three *Kurixalus* species (*K. odontotarsus*, *K. hainanus*, and *K. lenquanensis* Yu, Wang, Hou, Rao, & Yang, 2017) are recognized in Yunnan, China (Yu et al. 2017a, Yu et al. 2017b). The new species differs from *K. odontotarsus* and *K. hainanus* by having smaller ratio of head length to body size and no large dark spots on abdomen (versus larger ratio of head length to body size and large dark spots on entire abdomen; Figs 4, 9) and from *K. lenquanensis* by larger body size (SVL of 31.6–34.7 mm in adult males), more pointed snout, and presence of green coloration on dorsal surface and lateral side of head and body (versus smaller body size [SVL of adult males less than 30 mm], somewhat rounded snout, and absence of green coloration on dorsum; Fig. 9, Yu et al. 2017b).

The new species is distinguished from *Kurixalus idiootocus* (Kuramoto & Wang, 1987) by larger body size, absence of a pair of symmetrical large dark patches on chest, and single internal vocal sac (versus smaller body size [SVL of adult males less than 30 mm], presence of a pair of symmetrical large dark patches on chest, and single external vocal sac; Yu et al. 2017a); from *Kurixalus berylliniris* Wu, Huang, Tsai, Li, Jhang, & Wu, 2016 by gold brown irises, weak nuptial pads, and coarsely granular abdomen (versus emerald to light green irises, greatly expanded nuptial pads, and smooth abdomen; Wu et al. 2016); from *Kurixalus wangi* Wu, Huang, Tsai, Li, Jhang, & Wu, 2016 by larger body size, weak nuptial pads, and presence of supernumerary tubercles on foot (versus smaller body size [SVL of 28.6–31.6 mm in adult males], greatly expanded nuptial pads, and absence of supernumerary tubercles on foot; Wu et al. 2016); and from *Kurixalus eiffingeri* (Boettger, 1895) by weak nuptial pads, oblique loreal region, and curved canthus rostralis (versus greatly expanded nuptial pads, vertical loreal region, and straight canthus rostralis; Wu et al. 2016).

In addition, *Kurixalus yangi* sp. n. differs from *Kurixalus baliogaster* (Inger, Orlv, & Darevsky, 1999) by having serrated dermal fringes on limbs, tuberculate dorsum, tubercles on eyelids, and absence of large dark spots on venter (versus no serrated dermal fringes on limbs, dorsum smooth, tubercles on eyelids absent, and large dark spots scattered on entire venter; Inger et al. 1999); from *Kurixalus banaensis* (Bourret, 1939) by having larger body size and vomerine teeth (versus smaller body size [SVL of 26.2–33.2 mm in adult males] and vomerine teeth absent; Nguyen et al. 2014b, Bossuyt and Dubois 2001); from *Kurixalus viridescens* Nguyen, Matsui, & Duc, 2014 by having brown dorsal color, dark markings on dorsum and limbs, and vomerine teeth (versus uniformly greenish dorsal color with no dark markings on dorsum and limbs, and vomerine teeth absent; Nguyen et al. 2014a); and from *Kurixalus motokawai* Nguyen, Matsui, & Eto, 2014 by having larger body size and vomerine teeth (versus smaller body size [SVL of 23.2–28.4 mm in adult males] and vomerine teeth absent; Nguyen et al. 2014b).

*Kurixalus yangi* sp. n. can be distinguished from *Kurixalus ananjevae* Matsui & Orlov, 2004 by having vomerine teeth, serrated dermal fringes on limbs, and finely granular throat surface (versus vomerine teeth absent, serrated dermal fringes absent, and throat surface smooth; Matsui and Orlov 2004); from *Kurixalus verrucosus* (Boulenger, 1893) by granular throat and chest and interorbital space longer than upper eyelid (versus throat and chest smooth and interorbital space as broad as upper eyelid; Boulenger 1893); from *K. bisacculus* by having single internal vocal sac (versus paired external lateral vocal sacs; Taylor 1962); and from *Kurixalus appendiculatus* (Günther, 1858) by having rudimentary web between fingers, supernumerary tubercles, and outer metacarpal tubercle (versus one third web between fingers, supernumerary tubercles absent, and outer metacarpal tubercle absent; Günther 1858, Brown and Alcala 1994).

## Discussion

Species diversity of the genus *Kurixalus* seems to be underestimated, with at least five unnamed lineages in the *K. odontotarsus* species group, with the exception of the new species described here, remaining to be described according to our earlier work (Yu et al. 2017a; Fig. 1). Taxonomic confusion in the *K. odontotarsus* species group mainly involved *K. bisacculus*. Of the remaining five clades that might represent unnamed species, four (clades F, G, H, and K; Fig. 1) were placed in *K. bisacculus* (Stuart and Emmett 2006, Thy et al. 2010, Yu et al. 2010, Nguyen et al. 2014a, Nguyen et al. 2014b). Even *K. hainanus* (clade J) was considered a synonym of *K. bisacculus* (Yu et al. 2010). A reason for this situation is the relatively low divergence of 16S rRNA sequences between *K. bisacculus* and these clades, which resulted in these lineages being considered conspecific though morphological differences exist between them (e.g., Yu et al. 2010). Another source of taxonomic confusion in the *K. odontotarsus* species group involves *K. verrucosus*, as specimens from northern Myanmar (*Kurixalus* sp5; Fig. 1) and *Kurixalus naso* from southern Tibet (clade A, Fig. 1) had been wrongly treated as *K. verrucosus* in previous molecular studies (Yu et al. 2010, Yu et al. 2013, Li et al. 2013, Nguyen et al. 2014a, Nguyen et al. 2014b) according to Yu et al. (2017a). Additionally, with the exceptions of those unnamed lineages revealed by Yu et al. (2017a), cryptic species likely also exist in Philippine populations of *K. appendiculatus* according to Gonzalez et al. (2014). In short, combination of the two recent molecular studies based on broad sampling (Gonzalez et al. 2014, Yu et al. 2017a) has provided a relatively clear genetic framework for the taxonomy of *Kurixalus* and more morphological studies will be necessary to verify the specific status of those lineages.

Phylogenetically, the *K. odontotarsus* species group is comprised of two clades; one contains *K. yangi* sp. n., *K. naso*, and *K.* sp5 and one contains other species from Indochina and southern China (Fig. 1). *Kurixalus yangi* sp. n. is known from western Yunnan, China and northern Myanmar, *K. naso* is known from southern Tibet and northeastern India, and *K.* sp5 is known from northern Myanmar. This pattern suggests that frogs of *Kurixalus* might have colonized the Indian subcontinent from northern Indochina.

### Key to the new species and its congeners

**Table:** 

1	Limbs without serrated dermal fringes	**2**
–	Limbs with serrated dermal fringes	**3**
2	Dorsum smooth; many dark spots scattered on ventral surface	***K. baliogaster***
–	Dorsum with small tubercles, no dark spots on ventral surface	***K. ananjevae***
3	Dorsal color uniformly greenish	***K. viridescens***
–	Dorsal color not uniformly greenish, generally brownish mixed with dark marking	**4**
4	Iris emerald to light green	***K. berylliniris***
–	Iris golden	**5**
5	Nuptial pad greatly expanded	**6**
–	Nuptial pad slight	**7**
6	Tubercles on lateral margin of finger IV connected with dermal fringe; venter whitish with very little pigmentation; loreal region oblique; canthus rostralis curved	***K. wangi***
–	Tubercles on lateral margin of finger IV separated from each other; venter with numerous fine brownish dots, especially in the gular region; loreal region vertical; canthus rostralis straight	***K. eiffingeri***
7	Vomerine teeth absent	**8**
–	Vomerine teeth present	**9**
8	Snout tip less markedly pointed; lateral fringes on limbs and infra-cloacal tubercles less developed; lateral sides areolate	***K. motokawai***
–	Snout tip markedly pointed; lateral fringes on limbs and infra-cloacal tubercles developed; flanks smooth	***K. banaensis***
9	Smaller body size (adult male SVL less than 30 mm)	**10**
–	Bigger body size (generally adult male SVL greater than 30 mm)	**11**
10	Snout obtusely pointed with no prominence on tip; absence of a pair of symmetrical large dark patches on chest; single internal vocal sac	***K. lenquanensis***
–	Snout pointed with a small prominence on tip; a pair of symmetrical large dark patches present on chest; single external vocal sac	***K. idiootocus***
11	Snout rounded or somewhat pointed; chin and breast smooth	***K. verrucosus***
–	Snout obviously pointed; chin and breast granular	**12**
12	Paired external lateral vocal sacs	***K. bisacculus***
–	Single internal vocal sac	**13**
13	Outer metacarpal tubercles absent	***K. appendiculatus***
–	Outer metacarpal tubercles present	**14**
14	Ventral surface shaded posteriorly with dark spots	**15**
–	Whole ventral surface shaded with large dark spots	**16**
15	Longer head, snout, and limbs; interorbital distance narrower than internarial distance and upper eyelid width	***K. naso***
–	Shorter head, snout and limbs; generally interorbital distance wider than internarial distance and upper eyelid width	***K. yangi* sp. n.**
16	Omosternum unforked	***K. odontotarsus***
–	Omosternum forked	***K. hainanus***

### Comparative material examined

*Kurixalus naso*: KIZ 180001R–180003R (field number: Rao 06304–06306), KIZ 180004R (field number: Rao 06308), KIZ 1800005R (field number: Rao 06309), KIZ 180006R (field number: Rao 06311), Motuo, Tibet, China. The sampling locality of these specimens is close to the type locality of *K. naso* (Egar stream between Renging and Rotung, Motuo, Tibet, China [in area claimed by India]) and morphological evidence provided in Yu et al. (2017a) indicates that the clade consisting of these specimens (clade A; Fig. 1) is most likely *K. naso*.

*Kurixalus hainanus*: KIZ 180007R (field number: Rao 14111303), KIZ 180008R (field number: Rao 14111304), Diaoluo Mt., Hainan, China (type locality of the species); KIZ 180009Y–180011Y (field number: YGH 090266, YGH 090268, YGH 090269), Nanning, Guangxi, China; KIZ 180012Y–180015Y (field number: YGH 090201, YGH 090202, YGH 090204, YGH 090205), Longmeng, Guangdong, China. These specimens were grouped in clade J (Fig. 1; Yu et al. 2017a) and morphologically differ from *K. bisacculus* (where they were previously placed in synonymy) by having single internal vocal sac (versus paired external lateral vocal sacs; see Yu et al. 2017a).

*Kurixalus odontotarsus*: KIZ 180016Y–180023Y (field number: YGH 090130–090137), Caiyanghe, Puer, Yunnan, China. The sampling locality of these specimens is close to the type locality of this species (Mengyang, Jinghong, Yunnan; *ca.* 75 KM) and genetically these specimens were grouped together with *K. odontotarsus* from the type locality according to previous studies (clade D in Yu et al. 2017a, clade II in Yu et al. 2010).

## Supplementary Material

XML Treatment for Kurixalus yangi
